# Microbial desalination cell with sulfonated sodium poly(ether ether ketone) as cation exchange membranes for enhancing power generation and salt reduction

**DOI:** 10.1016/j.bioelechem.2018.02.004

**Published:** 2018-06

**Authors:** Francisco Lopez Moruno, Juan E. Rubio, Plamen Atanassov, José M. Cerrato, Christopher G. Arges, Carlo Santoro

**Affiliations:** a Department of Civil Engineering, University of New Mexico, Albuquerque, NM, USA; b Center Micro-Engineered Materials (CMEM), Department of Chemical and Biological Engineering, University of New Mexico, Albuquerque, NM, USA; c Cain Department of Chemical Engineering, Louisiana State University, Baton Rouge, LA 70803, USA; d Bristol BioEnergy Center, Bristol Robotics Laboratory, University of West England, Bristol, UK

**Keywords:** Microbial desalination cells, SPEEK cation exchange membranes, Desalination, Power generation

## Abstract

Microbial desalination cell (MDC) is a bioelectrochemical system capable of oxidizing organics, generating electricity, while reducing the salinity content of brine streams. As it is designed, anion and cation exchange membranes play an important role on the selective removal of ions from the desalination chamber. In this work, sulfonated sodium (Na^+^) poly(ether ether ketone) (SPEEK) cation exchange membranes (CEM) were tested in combination with quaternary ammonium chloride poly(2,6-dimethyl 1,4-phenylene oxide) (QAPPO) anion exchange membrane (AEM). Non-patterned and patterned (varying topographical features) CEMs were investigated and assessed in this work. The results were contrasted against a commercially available CEM. This work used real seawater from the Pacific Ocean in the desalination chamber. The results displayed a high desalination rate and power generation for all the membranes, with a maximum of 78.6 ± 2.0% in salinity reduction and 235 ± 7 mW m^−2^ in power generation for the MDCs with the SPEEK CEM. Desalination rate and power generation achieved are higher with synthesized SPEEK membranes when compared with an available commercial CEM. An optimized combination of these types of membranes substantially improves the performances of MDC, making the system more suitable for real applications.

## Highlights

• Thin and more conductive cation exchange membranes were employed in MDCs.
• CEMs with different topographical patterns were investigated.
• Maximum power achievement in MDC was 235 ± 7 mW m^−2^.
• Maximum desalination achieved was 78.6 ± 2% over 3 days operations.
• SPEEK CEM membranes outperformed commercial membranes.

## 1 Introduction

Worldwide demand of water supply is increasing every day due to different factors that include population growth and higher domestic demands in developing countries. As a result, ensuring a sustainable future water supply is a concern [1]. Drinking water resources primarily come from fresh surface waters, ground water extraction, and desalination treatment of seawater [2]. Fresh surface waters and ground waters are over exploited in many areas across the globe. This over exploitation leads to water scarcity in diverse regions that are arid or semiarid, have low precipitations, or have no access to great rivers [1,2]. Over exploitation in areas lacking abundant fresh water resources suffer from contamination, due to the high waste discharges and impossibility of self-clean by the natural systems. Hence, the combination of these problems results in poor-quality water from rivers and lakes fostering greater energy and economic costs for water treatment. 97% of water on the Earth is under salty water form mainly residing in the oceans while the remaining 3% counts as fresh water coming from surface water, ground water, and glaciers [1,2]. Since the 1960s, desalination technology has played an important role in supplying drinking water. The number of active desalination treatment plants has continued to increase significantly over the 50 years time period [3,4]. However, desalination water treatment plants are only extensively built in developed countries, especially in the ones in which the unique source of water available is seawater. The cost of operation is very high due to the large amount of energy utilized and materials such as membranes [5,6]. These challenges motivate continued research with the intent to make desalination technologies more affordable and sustainable (more energy efficient).

Compared to the traditional desalination technology such as reverse osmosis (RO) [7], electrodialysis (ED) [8], nanofiltration (NF) [9] and distillation [10,11], other technologies have emerged as potential sustainable and cost effect alternatives to the more established technologies. One of these technologies, is microbial desalination cell (MDC) – the subject of this report, which is a relatively new technology currently being explored at the laboratory level [[12], [13], [14]]. MDC brings in concert a combination of electrochemistry, microbiology, membrane science, and mass transfer principles for electric power generation, while simultaneously removing salt from water and treating wastewater [15]. The MDC system is composed of three different chambers: the first chamber is the anode compartment in which organic matter is used as fuel and is oxidized. The next chamber is the desalination compartment that contains salty water. The anode chamber and desalination chamber are separated by an anion exchange membrane (AEM). The desalination chamber was partitioned from the cathode chamber (third and last compartment) through a cation exchange membrane (CEM). At the anode chamber, electroactive bacteria electrochemically oxidize organics and pollutants. At the cathode chamber, oxygen is electrochemically reduced closing the circuit. The sodium and chloride ions contained within the desalination chamber transfer to the anode and cathode chamber through the selective, ion-exchange membranes – sodium ions through the CEM and chloride ions through the AEM.

MDC technology has to overcome several important problems in order to become more effective and competitive compared to existing desalination technologies. The main issues with MDC are: i) low desalination rate, ii) degradation of organic matter, and iii) poor electrochemical performance [[15], [16], [17], [18], [19], [20], [21]]. Low power generation is ascribed to the low anodic kinetics and the high cathodic activation overpotentials. Additionally, the presence of membranes causes ohmic losses leading to lower power output compared to microbial fuel cells (MFCs). Typically, MDC uses electrodialysis membranes that are thick and have low ionic conductivity causing high area specific resistances [22,23]. Furthermore, the large ohmic overpotentials caused by the electrodialysis membranes yield low desalination rates in MDC [12,20]. Other notable problems are membrane fouling and chemical degradation, but those topics are not addressed in this report. Previous studies have shown improvement in the bioelectrochemical system using iron-based cathode catalysts [[24], [25], [26]], different selective membranes [[27], [28], [29]], integrating supercapacitors electrodes [[30], [31], [32], [33]] or recirculating the solution used [27,34]. Making gains in membrane materials and electrocatalysts will enable MDC to obtain reasonable power generation and desalination rates, so it can be competitive with today's established technologies. For those reasons, future research in bioelectrochemical systems (BESs) in wastewater treatment should consider scaling up as a critical issue. In the case of MFCs systems, different configurations have been developed from lab-scales to higher volumes with examples of 20 L [35,36], 45 L [37], 72 L [38], 250 L [39] and up to a maximum of 1000 L [40]. However, MDC systems have not been scaled beyond 100 L pilot plant systems [41]. Ion exchange membranes strongly impact the electrochemical performance of MDC, because the membranes constitute a significant resistance contribution (i.e., the ohmic overpotential or ohmic loses) in the assembled cell affecting the overall power generation and desalination rate. Mitigating the membrane resistance can be achieved by adjusting its thickness, selectivity and ionic conductivity. Anion and cation exchange membranes, as well as bipolar membranes, were tested in different experiments with MDCs. The initial study of X. Cao in 2009 [19], using AEM (DF120, Tianwei Membrane) and CEM (Ultrex CMI-7000, Membranes International) was used as a base for further investigations with MDCs. Generally, the majority of the membranes used during MDCs investigation are commercially available membranes from Membranes International INC. New Jersey, USA (AEM AMI-7000 and CEM CMI-7000) [13,27,34,42]. These membranes are thick and do not demonstrate high ionic conductivity. There has not been a systematic study of how membrane attributes impact the Figures of Merit for MDC (e.g., power output and desalination rate).

In this work, the Figures of Merit for MDC were studied with laboratory made CEM – sulfonated sodium (Na^+^) poly(ether ether ketone) (SPEEK). The SPEEK CEMs prepared were flat (i.e., no topographical patterns) and with micropatterned topographical patterns that had varying periodic lateral feature sizes (20 μm, 33 μm, 40 μm, and 80 μm). Following up with our results obtained in a previous study using laboratory made AEMs in MDC [43], SPEEK CEMs were combined with a laboratory made non-patterned AEM, quaternary benzyl trimethylammonium chloride poly(2,6-dimethyl 1,4-phenylene oxide) (QAPPO). Our baseline data was collected with commercially available AEMs and CEMs from Membranes International Inc. Activated sludge and real seawater from Pacific Ocean were used as solution in the anodic and desalination chamber respectively. Electrochemical measurements and operating conditions such as pH and solution conductivity were monitored and reported.

## 2 Materials and methods

### 2.1 Microbial desalination cell (MDC) configuration

**Fig. 1:**
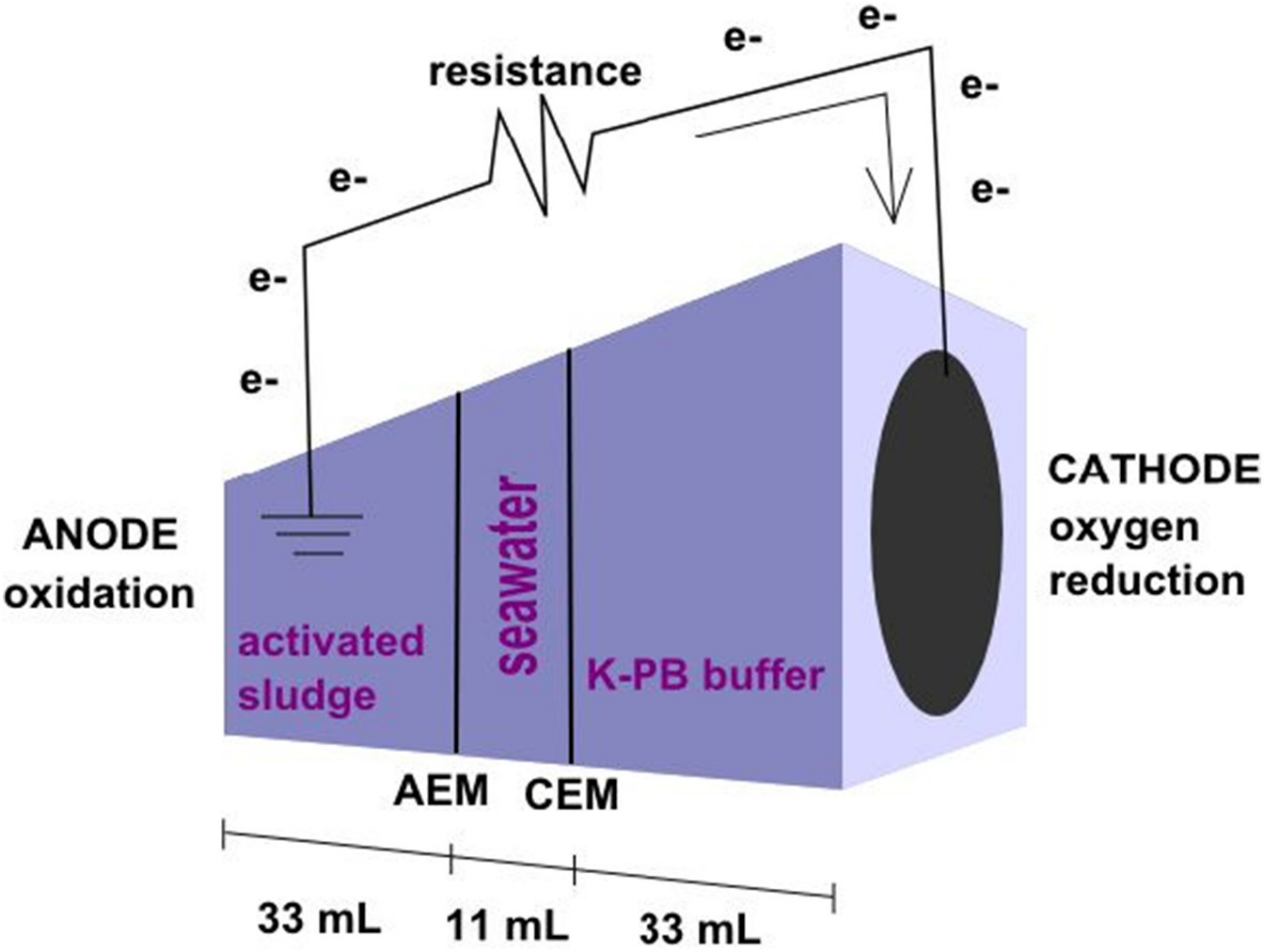
Schematic of microbial desalination cell set up used for this study.

The MDC consisted of three plastic compartments. Each electrode compartment was separated from the desalination chamber by a selective ion-exchange membrane (Fig. 1.a). The first chamber, the anode compartment, contained the anode electrode and it was filled with activated sludge obtained from the Albuquerque Southeast Water Reclamation Facility (Albuquerque, NM, USA) [44]. The same activated sludge was used for all experiments. 3 mL from a concentrated stock solution (100 g L^−1^) of sodium acetate was added as bacterial food. The empty volume of the anode chamber was 33 mL with a constant initial pH of 7.8 and it had a solution conductivity of 2.1 mS cm^−1^. The central chamber, labeled the desalination chamber, had 11 mL of volume, and was filled with real seawater (51.4 mS cm^−1^). The real seawater was collected in the Pacific Ocean specifically at Solana Beach - CA - USA. An anion exchange membrane (AEM) was positioned between the two chambers as physical separator. The AEM used in this work was a non-patterned QAPPO. The QAPPO was prepared via free radical bromination of poly(2,6-dimethyl 1,4-phenylene oxide) followed by nucleophilic substitution with trimethylamine and ion-exchange to the chloride form [45,46]. The third chamber assembled was the cathodic chamber with an empty volume of 33 mL and filled with a solution of 10 mM potassium phosphate buffer (K-PB) with pH of 7.8. In this case, the desalination chamber and the cathodic chamber were separated by the different cation exchange membranes (CEMs) tested in the experimentation (see section 2.3 for CEM preparation).

### 2.2 Electrodes used in microbial desalination cells

Anode and cathode electrodes were inserted in the anode chamber and cathode chamber respectively. The anode electrode was a carbon brush that had a cylindrical shape with diameter of 3 cm and height of 3 cm. Carbon brushes were built with carbon fibers wrapped on a titanium core (Millirose, USA). Before their use, each anode electrode was kept in a separate microbial fuel cell and the anode was already colonized with electroactive bacteria and well working before using the anodes for the MDCs experimentation [47,48]. The cathode electrode was designed in air-breathing configuration in order to have a three phase interface (TPI) and therefore be able to utilize oxygen in gas phase. New unused cathodes were fabricated and used during each cycle for consistency. The cathodes were based on activated carbon (AC), carbon black (CB) and polytetrafluoroethylene (PTFE) blended in a blender with a mixing ratio in weight of 8:1:2 of AC/CB/PTFE. The black powder obtained was inserted into a pellet die and then pressed over a stainless-steel mesh used as current collector through a hydraulic press at 2 mT for 5 min. The loading of AC/CB/PTFE for each cathode was 40 mg cm^−2^ and 7 cm^2^ of circular geometric area was exposed to the electrolyte [47,48]. Equal area of the cathode from the other side was exposed to the atmosphere.

### 2.3 Membrane materials: fabrication and characterization

Freestanding SPEEK CEMs were synthesized as reported in the literature [49]. Poly(arylene ether ether ketone) (PEEK) was dissolved in concentrated sulfuric acid (10 wt% in 98% pure sulfuric acid solution) and was mixed for 72 h at room temperature. The polymer was precipitated in an ice-cold deionized water bath and repeatedly washed and filtered until the pH of the washing water was 7. A 5 wt% SPEEK solution in *n*-methyl pyrrolidine (NMP) was prepared and the solution was drop casted on to 15 cm × 15 cm glass plate placed on a leveled surface in an oven. The oven temperature was then set to 70 °C and the solvent was evaporated over 18 h. The membrane on the glass plate was immersed in deionized water to remove it. Note: This is the flat SPEEK sample (S1). The resulting thickness of the membrane, after drying, was 30 μm. The SPEEK CEM was ion-exchanged to the sodium ion form by immersing the membrane in 1 M sodium hydroxide (NaOH) solution for 18 h followed by excessive rinsing and immersion in deionized water to remove excess salt.

**Fig. 2:**
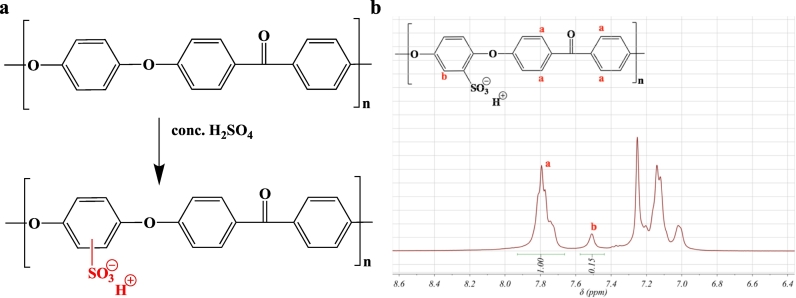
a.) Synthesis scheme to make SPEEK and b.) ^1^H NMR spectrum of prepared SPEEK.

The conversion of the base polymer, PEEK, to SPEEK was confirmed via ^1^H NMR spectroscopy using deuterated dimethyl sulfoxide (d-DMSO) solvent that contained tetramethylsilane (TMS) as an internal standard. The NMR spectrometer was a 400 MHz Bruker instrument. The amount of sulfonate groups per repeat unit (the degree of functionalization (DF)) was determined by integrating the ^1^H NMR spectrum (see Eq. (1)). Fig. 2.a gives the chemical reaction for converting PEEK into SPEEK and Fig. 2.b is the ^1^H NMR spectrum.

(1) $$\mathrm{DF}=\frac{{\mathrm{Area}}_{a}}{{\mathrm{Area}}_{b}}$$

**Fig. 3:**
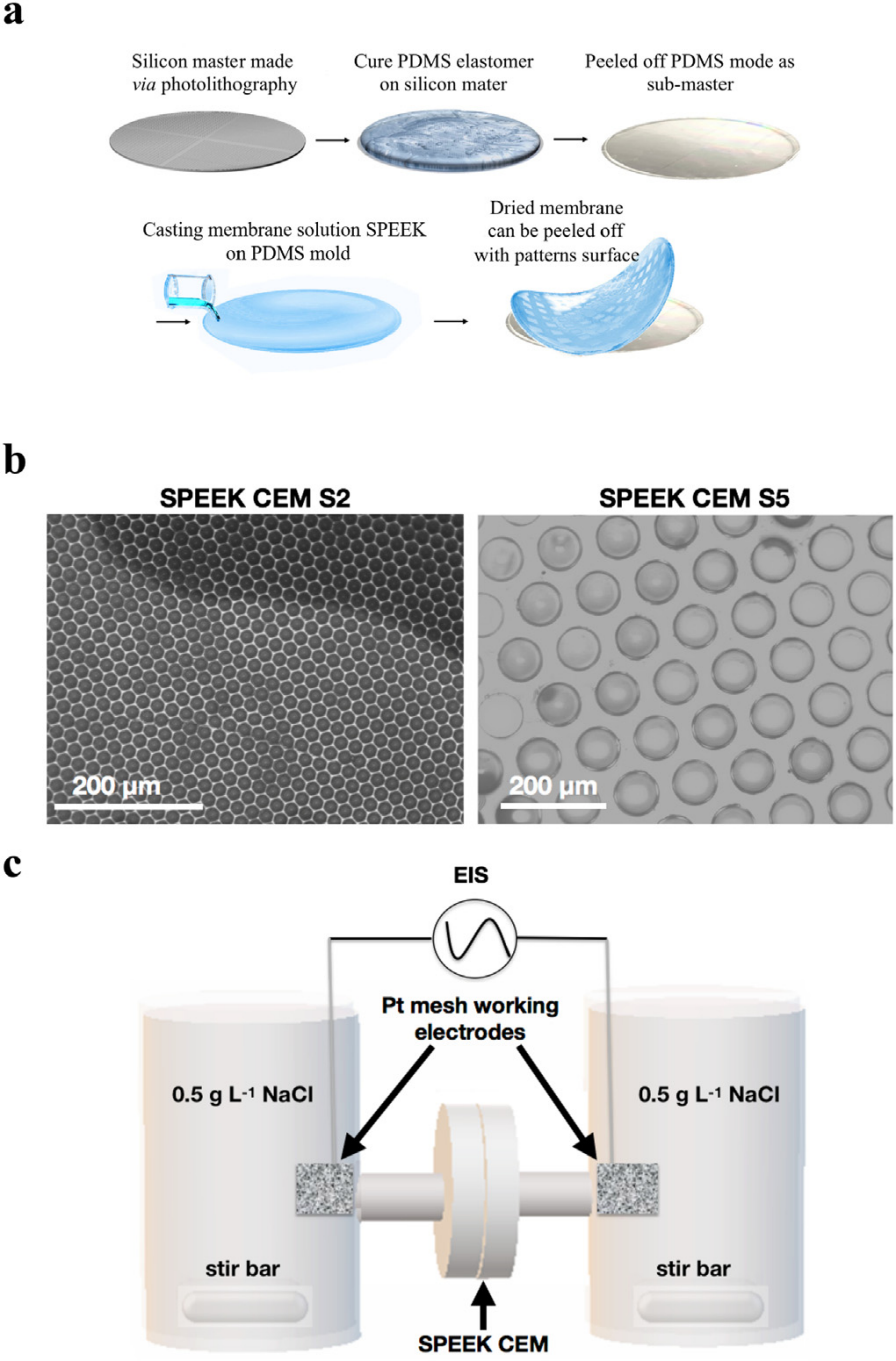
a.) Process flow to make micropatterned PDMS molds that are used for preparing topographically patterned SPEEK CEMs; b.) Optical micrograph images of SPEEK CEM S2 and S5 samples; c.) Concentration cell to measure the through-plane resistance and ionic conductivity for the SPEEK CEMs.

The SPEEK CEMs with different periodic, topographical patterns were prepared by drop casting the dissolved SPEEK solution in NMP on to micropatterned poly(dimethyl siloxane) (PDMS) molds that were prepared through conventional soft lithography as described in our previous report [50]. The different lateral feature sizes of the patterned SPEEK CEMs were: 20 (S2), 33 (S3), 40 (S4), and 80 (S5) μm). Fig. 3.a depicts the general scheme to create SPEEK CEMs with topographical patterns. The micropatterned SPEEK membrane surfaces were imaged with a Nikon OPTIPHOT-88 Optical Microscope. Fig. 3.b shows optical micrographs of two of the micropatterned SPEEK CEMs with different topographical lateral feature sizes.

The in-plane ionic conductivity of the SPEEK CEMs was determined by electrochemical impedance spectroscopy (EIS) using a 4-point platinum conductivity probe in deionized water and 0.5 g L^−1^ sodium chloride (NaCl). EIS, in galvanostat mode, was performed with a 2 mA amplitude in the frequency range of 100,000 Hz to 0.1 Hz. The in-plane resistance was determined from the Bode plot, where the resistance value had a phase angle value of zero, and was used in Eq. (2) to determine the in-plane ionic conductivity (σ).

(2) $$\sigma =\frac{L}{R\times t\times w}$$

where σ was the in-plane conductivity, R was the in-plane membrane resistance, t was the membrane thickness (fully hydrated membrane) and w was the membrane width (fully hydrated membrane).

The through-plane ionic conductivity of SPEEK CEMs was determined using a concentration cell with 6 g L^−1^ of NaCl solutions in each compartment (the lower concentration value expected in the MDC). The solutions were agitated with magnetic stir bars. The active area for the cell was 2 cm^2^. Each cell contained a platinum mesh working electrode (see Fig. 3.c). The resistance between the two working electrodes was measured with and without membranes using EIS in galvanostat mode (0.5 mA amplitutde in the frequency range of 100,000 Hz to 0.1 Hz). The resistance was determined from the Bode plot, where the resistance value had a phase angle value of zero. The through-plane membrane resistance (*R*_*m*_) was determined by subtracting the measured solution-membrane resistance (*R*_*m–s*_) in the concentration cell minus the resistance of the supporting electrolyte (*R*_*s*_ - i.e., no membrane in the cell) [51] – see Eq. (3).

(3) $$R_{m}\left[\Omega -{\mathrm{cm}}^{2}\right]=\left(R_{m-s}-R_{s}\right)∙\mathrm{ActiveArea}$$

### 2.4 Measurements

#### 2.4.1 Solution conductivity and pH

Solution conductivity and pH were measured initially and at 24 h intervals during each cycle. An instrument Omega PHB- 600R (Omega Engineering Inc., Norwalk, CT, USA) was used to record pH. Solution conductivity was recorded using an instrument Orion Star 112 Conductivity Meter (ThermoFisher Scientific. Waltham, MA, USA). Both instruments were calibrated prior to the use.

#### 2.4.2 Electrochemistry

Three days cycle (96 h) data was recorded by triplicate for each of the CEMs membranes and the commercial membrane, using the same setup and operating conditions, each cell was connected during the cycle to an external resistance of 470 Ω. Table 1 lists the membrane configurations tested in the MDC. At the end of each cycle (after 96 h), the three chambers were filled with new electrolytes in order to have identical operating conditions for all the MDCs working with the different membranes and polarization curves were measured. In order to collect polarization curves to obtain the power curves, two potentiostats Gamry Reference 600+ (Gamry Instruments, PA, USA) were utilized and linear sweep voltammetries (LSVs) were run. The first potentiostat was operating from open circuit voltage (OCV) and 0 mV at a scan rate of 0.2 mV s^−1^. Particularly, the working channel was connected to the cathode, the counter channel was connected to the reference Ag/AgCl (3 M KCl), with the reference channel was short circuited to the counter channel. In parallel, the second channel was recording the cathode potential during the LSV. Particularly, the working channel was connected to the cathode, the counter channel was connected to the anode and the reference channel was short circuited to the counter channel. Ag/AgCl (3 M KCl) was used as reference electrode and it was located into the desalination chamber. For both polarization and power curves, current and power are expressed as density values, referred to the cathode geometric area (7 cm^2^) for cathode that is actually the same area as the AEM, and CEM.

**Table 1:** In-plane conductivity and through-plane resistance of SPEEK CEMs in different liquid solutions.

Sample	In-plane ionic conductivity (mS cm^−1^)			Through-plane resistance (Ω-cm^2^)
	DI H_2_O at 20 °C	DI H_2_O at 40 °C	0.5 g L^−1^ NaCl at 20 °C	6 g L^−1^ NaCl at 20 °C
SPEEK CEM S1 - flat	2.9	10.7	320	23
SPEEK CEM S2 - 20 μm	n/a	6.5	291	38
SPEEK CEM S3 - 33 μm	n/a	8.2	288	28
SPEEK CEM S4 - 40 μm	n/a	6.6	290	23
SPEEK CEM S5 - 80 μm	3.7	5.6	333	24
Membranes International CMI-7000 CEM [52]	n/a	n/a	n/a	30^⁎^ ^⁎^Note: 30 g L^−1^

Note: *Data from the supplier [52]. All CEMs' counterions are the sodium ion. The measured resistance for the 0.5 g L^−1^ NaCl solution for the concentration cell (for through-plane resistance measurements) was 1061 Ω-cm^2^. The in-plane resistance for 0.5 g L^−1^ NaCl (with no membrane) was 262 Ω (9.5 mS cm^−1^). The supporting electrolyte conductivity was selected from the in-plane conductivity of SPEEK CEMs in 0.5 g L^−1^ NaCl. n/a – the in-plane impedance, which is used to calculate the ionic conductivity, of the SPEEK CEMs was quite large under deionized water in the sodium counterion form at 20 °C. Therefore, testing whether or not the topographical patterns impacted ionic conductivity of the CEM was tested at elevated temperatures to reduce the impedance and it was also tested with supporting electrolyte (0.5 g L^−1^) because it also reduced the impedance. Plus, testing the membrane resistance/ionic conductivity of the SPEEK CEMs in supporting electrolyte rather than deionized water is more representative of the conditions in the MDC.

## 3 Results and discussion

### 3.1 Membranes characterization

The ^1^H NMR in Fig. 2.b confirmed successful incorporation of sulfonic acid moieties into the PEEK polymer to make SPEEK, because a peak was detected at 7.5 ppm. The degree of sulfonation was 0.6 (i.e., the number of sulfonate groups per repeat unit) and that translated to an ion-exchange capacity (IEC) of 1.8 m mol g^−1^. The 10 g batch of SPEEK synthesized was used to make all patterned and non-patterned SPEEK CEMs. The optical micrograph images in Fig. 3.b verify the successful fabrication of periodic, topographical patterned features on the SPEEK CEMs. Table 1 reports the in-plane ionic conductivity of the SPEEK CEMs in deionized water at different temperatures (20 °C and 40 °C) and in supporting electrolyte (0.5 g L^−1^ NaCl). Additionally, Table 1 provides the through-plane resistance of the SPEEK CEMs. The in-plane ionic conductivity values showed high ionic conductivity values (296 to 342 mS cm^−1^) in a dilute supporting electrolyte (0.5 g L^−1^). This concentration of NaCl solution is substantially lower than the range of NaCl solutions experienced in the MDC (6 to 30 g L^−1^). There was no trend between micropatterned lateral feature size and SPEEK CEM ionic conductivity and through-plane resistance. It was hypothesized that patterning the CEM surface would increase the interfacial surface area between the membrane and the salt water in the desalination chamber. Having an increased interfacial area was anticipated to enhance the rate of salt uptake, which should manifest a lower ohmic resistance and a higher cell power density and greater salt removal. However, the patterned membranes did not produce a MDC with greater power density or salt removal when compared to the flat (i.e., non-patterned) CEMs. It is important to point out that the through-plane resistance and the in-plane resistance, characterized externally for the CEMs, was equivalent or worse with the patterned membranes. We ascribe the unexpected results to the following possibilities: i.) the patterned membranes trap small amount of particles or precipitates that hinder sodium ion transport and ii.) the micro-confined domains change the interface between the membrane and water slowing down the sodium ion migration. Similar results were observed for patterned and non-patterned AEMs in our previous study with MDC [43]. The impetus for using micropatterned ion-exchange membranes came from other reports showing that these materials enhance the performance of proton exchange membrane fuel cells (PEMFC) with hydrogen [53]. However, that system is different than the MDC because the interface for the PEMFC is a membrane-porous air cathode and here the interface is a membrane-water solution.

The flat SPEEK CEMs, in most cases, gave the highest in-plane ionic conductivity values and lowest through-plane resistance. It will be shown later that this membrane yielded the highest power output and desalination rate for the MDC indicating the patterned features did not provide any significant gains for the MDC – a same observation seen in our previous report for MDC with micropatterned AEMs [43]. Finally, it should be noted that all of the SPEEK CEMs had a lower through-plane resistance than the Membranes International CEM (data reported by the manufacturer) [52]. The Membranes International CEM was tested in a more concentrated supporting electrolyte when compared to our tests (approximately 30 g L^−1^ (0.5 M) NaCl). Because the SPEEK CEMs' resistance in 30 g L^−1^ NaCl was so low (on the order of 8 Ω-cm^2^), the difference between the membrane-solution and solution resistance was almost zero - i.e., the membrane contribution to resistance could not be detected. The lower through-plane resistance and higher ionic conductivity of the SPEEK CEMs, in addition to being thinner (50 μm versus 450 μm for the Membranes International CEM), indicated that these membranes were good candidates to lower the ohmic overpotential for the MDC.

Membrane ionic conductivity and thickness can be combined to calculate the area specific resistance (ASR) as shown below in Eq. 4. Note that the units for ASR are ohm-cm^2^ (or cm^2^ S^−1^). In this equation, higher ionic conductivity yields a lower ASR. A thinner membrane also gives a smaller ASR. A membrane with both high ionic conductivity and a small thickness value work synergistically to drastically reduce the ASR. Reducing all the resistances within the MDC maximizes the power output and the desalination rate. A smaller ASR for both the AEM and CEM is critical for improving the thermodynamic efficiency and desalination performance of the MDC.

(4) $$\mathrm{ASR}=\frac{L}{\kappa }$$

L = membrane thickness.

κ = membrane ionic conductivity.

### 3.2 Power curves

**Fig. 4:**
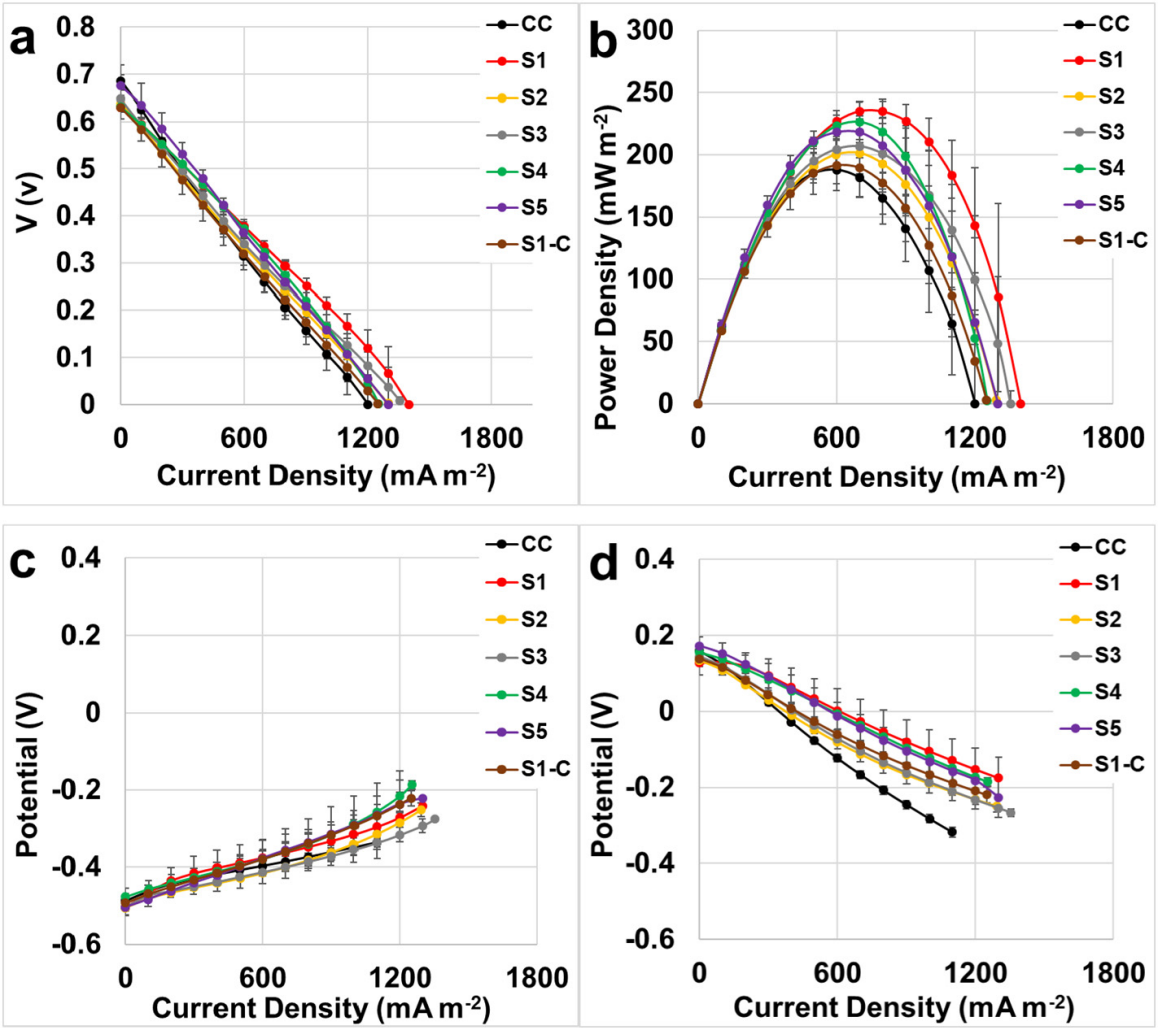
Overall polarization curve (a), power curves (b), anode (c) and cathode (d) polarization curves of the MDCs having different CEMs.

MDCs were tested keeping the same AEM, in this case QAPPO, and changing the CEM among the previously described SPEEK membranes [43]. The electrochemical results are displayed in Fig. 4 and particularly, polarization curves (Fig. 4.a), power curves (Fig. 4.b), and anode (Fig. 4.c) and cathode (Fig. 4.d) polarization curves were obtained. These curves were recorded after anode and cathode solutions were replenished after the third day cycle in order to have identical operating conditions.

Initial open circuit voltage (OCV) of the MDCs showed as initial point (null current density) of the overall polarization curve (Fig. 4.a) was similar for all the MDCs and quantified in 0.65 ± 0.02 V. This value was independent of the membrane utilized. At short circuit current density, the utilization of commercial CEMs (CC) recorded the lowest value of 1200 mA m^−2^. In parallel, a maximum short circuit current of 1399 mA m^−2^ was measured when S1 membrane was used as CEM. The short circuit average currents and the standard deviations (based on *n* = 3 measurements) obtained for each membrane were 1200 ± 1 (CC), 1363 ± 52 (S1), 1293 ± 5 (S2), 1355 ± 63 (S3), 1253 ± 52 (S4), 1263 ± 55 (S5) and 1250 ± 60 (S1-C) mA m^−2^. The linear trends observed in the polarization curves highlight that MDC power output is governed by ohmic losses for all cases. These results suggest that future efforts should be geared towards minimizing ohmic overpotentials in MDC.

The power curves were calculated from polarization data according to the following equation: P = I × V (Fig. 4.b). MDCs with membrane S1 (non-patterned) recorded the highest power density 235 ± 7 mW m^−2^ at a current density of ≈700 mA m^−2^. This result is ≈20% better than the best outcomes obtained in previous MDC study in which QAPPO was used as anion exchange membrane and commercial CEM [43]. Combination of both commercial anion and cation exchange membrane reached 188 ± 11 mW m^−2^ at a current density of 600 mA m^−2^, which was 20% lower in power density when compared to S1. The MDCs having different membranes had a peak of power density of 201 ± 19 mW m^−2^, 204 ± 16 mW m^−2^, 226 ± 16 mW m^−2^ and 218 ± 13 mW m^−2^ for S2, S3, S4, and S5 respectively. These results are very similar and all below the S1 outcome. As observed in our previous study [43], the topographical patterns with different lateral sizes did not generate enhance power generation.

The anode (Fig. 4.c) and cathode (Fig. 4.d) polarization curves were obtained inserting the reference electrode in the central chamber and recording the potential variation during the polarization curve. The analysis of the anodic data sets shows similar trends for all the membranes, which was expected because the same identical membrane and the high-performing anode electrode was used. Negligible differences in potential (max of 40 mV) were detected at 600–700 mA m^−2^ in which the maximum power generations were recorded; therefore, the differences in power curves was attributed to the cathode. Considering the cathode polarization curves (Fig. 4.d), different slopes in the trends were noticed for every different membrane utilized. The slope of the curve was ascribed to the ohmic losses, because identical cathodes materials and the same solution was used during the overall polarization curves. Hence, the higher resistance was related to the different membranes studied. The polarization curves revealed that S1 had the lowest ohmic resistance, while the CC had the highest ohmic resistance. These results demonstrate that reducing the membrane resistance lowered MDC polarization leading to greater power output.

### 3.3 Desalination

**Fig. 5:**
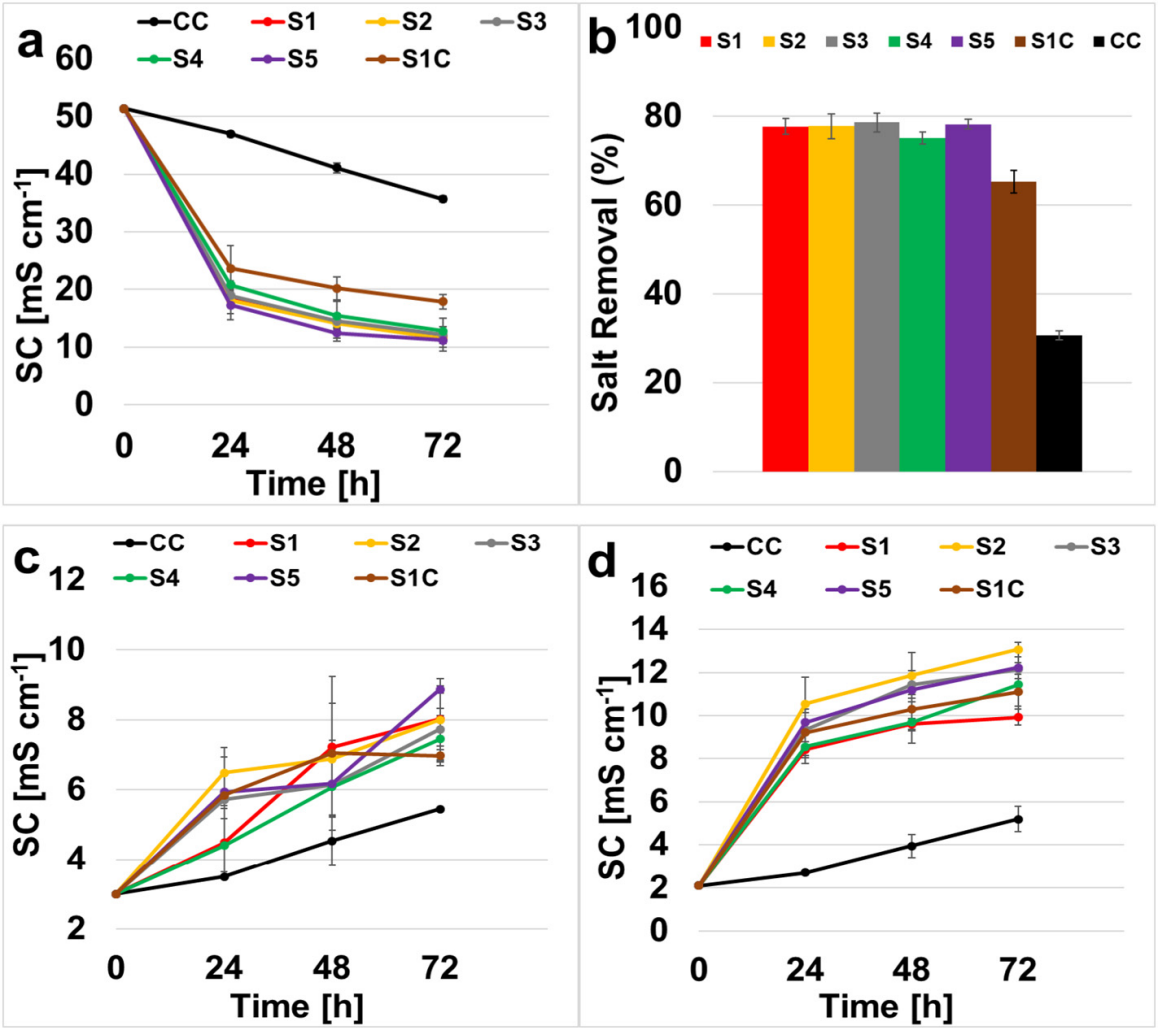
Desalination chamber solution conductivity (a), desalination chamber salt removal (b), anode chamber solution conductivity (c), cathode chamber solution conductivity (d).

The initial solution conductivity for the seawater placed in the desalination chamber at the start of each experiment was 51.4 mS cm^−1^. The results displayed a final solution conductivity that was very similar and corresponded to 11.4 ± 0.9 mS cm^−1^, 11.4 ± 1.4 mS cm^−1^, 11 ± 1 mS cm^−1^, 12.8 ± 0.7 mS cm^−1^, 11.2 ± 0.5 mS cm^−1^ for the utilization of membrane S1, S2, S3, S4 and S5 respectively (Fig. 5.a). This corresponded to a reduction in salinity content of 77.7 ± 1.8%, 77.7 ± 2.7%, 78.6 ± 2%, 75 ± 1.4% and 78.2 ± 1.1% respectively (Fig. 5.b). Generally speaking, the results did not show relevant differences between patterned and non-patterned membranes indicating that the lateral sizes did not play a major role into the desalination. These amounts are much higher than the recorded values by the combination of commercial membranes, which displayed a 30.6 ± 1% in terms of removal salt, with a final 35.7 ± 0.5 mS cm^−1^. These results with SPEEK gave a 25% improvement in terms of salt removal respect to the results obtained in the previous study using combination of commercial CEM and QAPPO AEM [43].

The solution conductivity at the anode chamber (Fig. 5.c), that had an initial point of 2.1 mS cm^−1^, showed a more variable picture with values that ranged between 7 mS cm^−1^ and 9 mS cm^−1^. The lowest value recorded of 5.45 mS cm^−1^ was measured when a commercial anion exchange membrane was used. The trend was always increasing indicating a transport of negative ions from the desalination chamber to the anodic chamber. The cathode chamber was filled with the same buffer solution with initial solution conductivity of 2.1 mS cm^−1^ as start point. The increasing trend in solution conductivity was very similar for all the SPEEKs membranes reaching a maximum range between 10 mS cm^−1^ and 13 mS cm^−1^ that was 5 to 6-fold the initial value (Fig. 5.d). A smaller increase, up to 4.6 mS cm^−1^, was measured for the commercial membrane, because this membrane transferred fewer ions.

### 3.4 pH variation

**Fig. 6:**
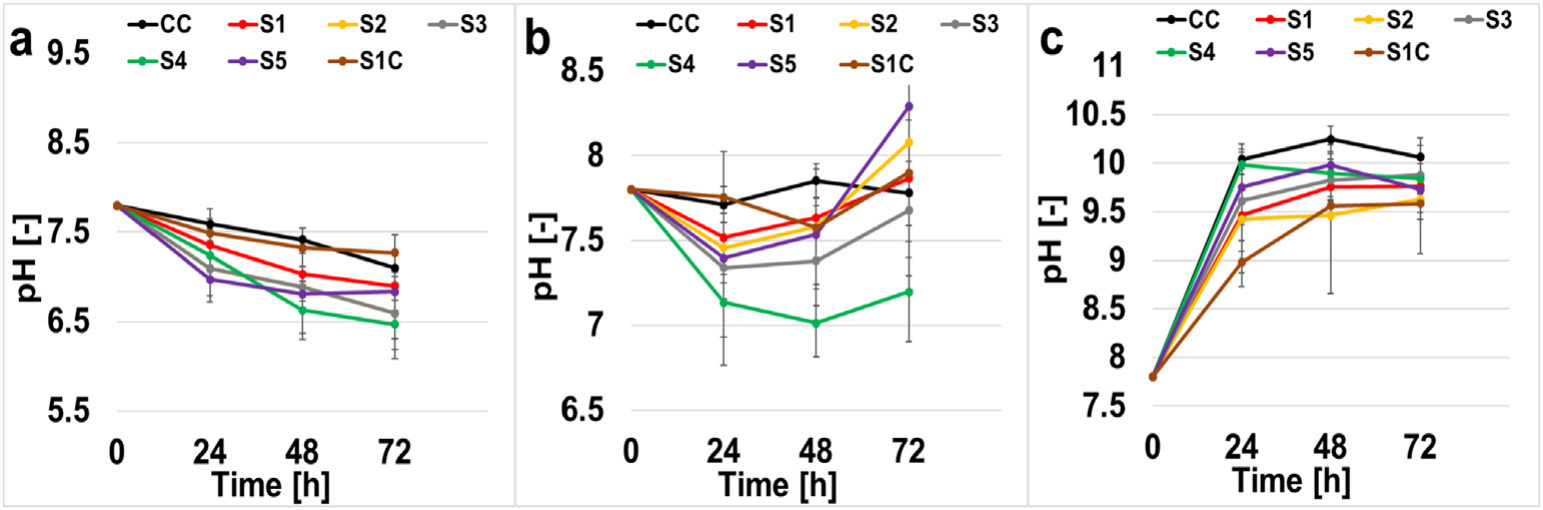
Anode chamber pH (a), desalination chamber pH (b), cathode chamber pH (c).

The pH was another important parameter that was monitored over time. Activated sludge taken from the same existing batch was used in each cell for the anode chamber, with an initial pH of 7.8 (Fig. 6.a). This initial value decreased up to 6.8 ± 0.2 for all SPEEKs membranes, and up to a lower value of 7.1 ± 0.1 for the commercial membrane. This decrease might be explained by the increase of H^+^ concentration as a product of the oxidation of organics, leading to an acidification of the media. In the case of cathode chamber (Fig. 6.b), the initial buffer pH was also 7.8, but inversely here, the values displayed incremented up to 9.81 ± 0.15. This value was very similar for all the cells independently from the membrane utilized.

This can be attributed to the products of the oxygen reduction reaction (ORR) produced at the cathode. In fact, the reaction at the cathode can proceed two different directions in function of the working electrolyte (e.g. acidic or alkaline). As the reaction occurs in acidic media, H^+^ is consumed and water is produced. In parallel, if the reaction takes place in alkaline media, OH^–^ is the final product. Both ORR pathways lead to the alkalization of the cathode chamber over time and this can be attributed to i) the consumption of H^+^ or ii) to the production of OH^−^.

The desalination chamber (Fig. 6.c) that was filled with the seawater had an initial pH value of 7.8, showed a more stable trend ending in a range between 7.4 and 8. This stability was probably due to the absence of electrochemical reactions occurring in this specific chamber.

### 3.5 Membrane long term performance and cost

The CEMs are anticipated to be stable for the long-term as the sodium chloride solution in the desalination chamber is benign. The CEM does interface with the air cathode and oxygen reduction can yield reactive oxygen species (ROS). The polyaromatic nature of the SPEEK backbone will make it resistant to oxidation by ROS. The ROS expected in the catholyte will be superoxide as this species is favored under alkaline conditions [54,55]. Strong oxidizing agents like hydroxyl and hydroperoxyl radicals, formed from the decomposition of hydrogen peroxide (parasitic product from oxygen reduction), are favored under acidic conditions [56,57]. The steady-state pH of the catholyte chamber of the MDC is 9.5 to 10 supporting a basic environment in the catholyte chamber. Therefore, the polyaromatic nature of the SPEEK and absence of hydroxyl and hydroperoxyl radicals suggest that the CEM will be stable for extended periods of time. Future efforts will need to examine SPEEK stability in the presence of superoxide species.

The membrane costs are quite low compared to membranes sold on the market. These are prepared from low cost and abundant commercially available poly(arylene ether) polymers using simple and straightforward reactions. Recently the price of these membranes is estimated at $198 per m^2^, but through scale-up, the membranes based upon the poly(arylene ether) polymers can be priced as low as $2 per m^2^ [58]. Electrodialysis membranes by Tokuyama (industry leader), quoted from Ameridia – a supplier for Tokuyama, are $356 per m^2^.

## 4 Outlook

In this work, the utilization of laboratory made anion and cation exchange membrane led to an increase in desalination rate and power generation in MDC. The results in terms of power generation are still lower than the ones existing in literature [27]. However, in terms of desalination, the results are much closer to the existing reported values and in many cases, even better than the results obtained in other studies with similar MDC systems, taking into account the utilization of synthetic salt waters with initial solution conductivity values of 30–35 mS cm^−1^ [12,20,59,60]. The reduction of dissolved salt in the desalination chamber over time causes an increased resistance from this chamber over time. This is often seen in electrodialysis and reverse electrodialysis in which the dilute chamber is the biggest source of resistance [61]. One strategy to combat this problem is to load a porous bed into the desalination chamber that conducts ions but does not add ions to the liquid phase, using a similar approach to that for electrodeionization [62]. However, a porous resin-wafer [63] is more effective than a packed column that is commonly used in electrodeionization. The maximum power achieved in this work was 235 ± 7 mW m^−2^ and the highest desalination rate was roughly 80% after 3 working days.

Lower performances compared to existing literature can be attributed to the limitations in the current experiments due to the low operating temperature (room temperature of 22 ± 2 °C) [64,65]. It was previously shown that low temperatures hinder the anode oxidation reactions kinetics. Moreover, in this work, real solutions were used such as activated sludge on the anode chamber with a low solution conductivity of 2.1 mS cm^−1^. Once again, it was shown that low solution conductivity affects negatively the performances [66,67].

From our results, we are encouraged to continue our efforts to improve the membranes for MDC. Lowering the resistance will still be a priority in addition to enhancing the chemical and physical stability (i.e., mitigating following) so they can operate effective for long time use and many cycles. Additionally, we plan on testing these membranes with flow recirculation in order to optimize the life cycle, for a possible scale up of the system.

## 5 Conclusions

Utilizing thinner and more conductive AEMs and CEMs, prepared by functionalizing commercially available polymers with ionic groups using facile and established procedures, enhanced the power output and desalination rate for MDC when compared to baseline studies that employed thick AEMs and CEMs that have low ionic conductivity. The maximum power generation achieved during this investigation was 235 ± 7 mW m^−2^. Solution conductivity decreased by 60% within the first 24 h and up to 80% after 3 days substantiating the desalination process. The pH increased above 9.5 after 24 h due to the alkalization of the cathode. Membranes with non-patterned surfaces outperformed membranes with different topographical patterns of varying lateral feature sizes. The ionic conductivity of the flat membranes was slightly higher than the patterned membranes and is the reason why the flat membranes yielded the best power output and desalination rate. Hence, the added processing of patterning membranes to increase greater interfacial area between the liquid solution and the membrane to reduce interfacial charge-transfer resistance did not occur as hypothesized.
